# Recent applications of stimulus-responsive smart hydrogels for osteoarthritis therapy

**DOI:** 10.3389/fbioe.2025.1539566

**Published:** 2025-02-17

**Authors:** Zhuoming Xu, Jintao Liu, Hanyin Hu, Jun Ma, Haiyang Yang, Jiayi Chen, Hongwei Xu, Haodong Hu, Huanhuan Luo, Gang Chen

**Affiliations:** ^1^ Jiaxing University Master Degree Cultivation Base, Zhejiang Chinese Medical University, Hangzhou, China; ^2^ Department of Orthopaedics, Jiaxing Key Laboratory of Basic Research and Clinical Translation on Orthopedic Biomaterials, The Second Affiliated Hospital of Jiaxing University, Jiaxing, China

**Keywords:** osteoarthritis, articular cartilage, responsive hydrogel, drug delivery, biomaterial application

## Abstract

Osteoarthritis is one of the most common degenerative joint diseases, which seriously affects the life of middle-aged and elderly people. Traditional treatments such as surgical treatment and systemic medication, often do not achieve the expected or optimal results, which leads to severe trauma and a variety of side effects. Therefore, there is an urgent need to develop novel therapeutic options to overcome these problems. Hydrogels are widely used in biomedical tissue repairing as a platform for loading drugs, proteins and stem cells. In recent years, smart-responsive hydrogels have achieved excellent results as novel drug delivery systems in the treatment of osteoarthritis. This review focuses on the recent advances of endogenous stimuli (including enzymes, pH, reactive oxygen species and temperature, etc.) responsive hydrogels and exogenous stimuli (including light, shear, ultrasound and magnetism, etc.) responsive hydrogels in osteoarthritis treatment. Finally, the current limitations of application and future prospects of smart responsive hydrogels are summarized.

## 1 Introduction

Osteoarthritis (OA) is a common degenerative joint disease that harms the health of many elderly people (Barnett, 2018; Hu et al., 2021; Yin et al., 2023). It is characterized by cartilage degeneration and local aseptic inflammation (Chen et al., 2024; Li Z. et al., 2024; Sun et al., 2024). The occurrence of osteoarthritis may be related to age, obesity, genetic, endocrine and metabolic diseases, inflammation, trauma and other factors (Palazzo et al., 2016; Berenbaum et al., 2018). Clinically, osteoarthritis patients have many symptoms such as joint pain, local deformity and mobility dysfunction, which seriously affect the life of patients (Abramoff and Caldera, 2020; Hall et al., 2022). According to the degree of lesion of OA, the pathophysiology usually divides it into three stages: early stages, middle stages and advanced stages (Martel-Pelletier et al., 2016; Jiang, 2022; Kalairaj et al., 2024). In the early stages of OA, the pathological changes inside the joint are mainly concentrated in the synovial region, manifested as synovial hyperplasia and fibrosis. At the same time, pro-inflammatory cytokines (also known as inflammatory mediators, such as IL-1β, IL-6, 15, 17, 18, 21, 22, and TNF-α) are increased in joint cells (including chondrocytes, osteoblasts, and synovial fibroblasts), and activate related inflammatory signaling pathways, for example, the pathogenic mechanism of IL-1β is mainly related to its binding to IL-1 receptor I (IL-1RI). When a large amount of IL-1β binds to the receptor, it will activate the mitogen-activated protein kinase (MAPK) pathway and the signaling pathway mediated by nuclear factor kappa-B (NF-κB), and induce the expression of proteases related to chondrodecomposition such as MMP (Matrix metalloproteinase) and ADAMTS (A disintegrin and metalloproteinase with thrombospondin motifs), which causes a lot of inflammation in the synovial area (Li et al., 2021a; van den Bosch, 2021; Liew et al., 2023), and gradually begin to stimulate cartilage breakdown (Ng et al., 2023). However, due to the mild early symptoms of OA, it is difficult for patients to detect and pay attention to it, and thus fail to carry out treatment in time, which will cause OA to progress to the next stage (Mahmoudian et al., 2021). In the middle stages of OA, inflammation inside the joints and synovium become more severer, and the lesion gradually spread to the cartilage, showing degeneration of the chondrocyte extracellular matrix, fibrosis in the calcified area, and cell hypertrophy (Wang et al., 2022). Due to the increase of a large number of inflammatory factors and the activation of inflammatory pathways such as NF-κB, MAPK and Wnt, a large number of proteases related to chondrolysis, such as MMP and ADAMTS, are continuously released from the diseased chondrocytes, aggravating cartilage injury and degeneration (Malemud, 2019; Li T. et al., 2022). At this stage, patients will feel obvious pain and discomfort in the joint. If OA progresses to an advanced stage, the lesion area extends to all joints, presenting with abnormal bone remodeling and hardening. Among them, transforming growth faction-β (TGF-β) in the cartilage microenvironment can be activated to accelerate the invasion of blood vessels inside cartilage, and accelerate angiogenesis by stimulating mesenchymal stem cells (MSCs), resulting in a large number of blood vessels of subchondral bone passing through calcified cartilage and invading cartilage, promoting chondrocyte hypertrophy and abnormal proliferation of bone tissue (Rim et al., 2020; Yao et al., 2023). Finally, the patient has a large number of osteophytes in the joint, producing severe pain and severe limitation of movement (Yan et al., 2022).

However, the traditional treatment of OA aims to relieve the clinical symptoms of pain and improve joint function (Mandl, 2019). The methods mainly include non-drug treatment, such as the improvement of lifestyle habits, massage physiotherapy and functional exercise, which are generally suitable for patients in the early stage of OA (Bannuru et al., 2019; Bennell and Hunter, 2020; Ni et al., 2024). Medications commonly used include nonsteroidal anti-inflammatory drugs (NSAIDs), opioid analgesics, steroid hormones, and hyaluronic acid (HA) (Richard et al., 2023), which are employed through oral administration, intravenous administration, articular injection and transdermal administration (Jones et al., 2019). However, drugs can only slow down the process of degeneration, which can not effectively promote the repair and regeneration of articular cartilage (Ma L. et al., 2022). In addition, the drug is in poor bioavailable by oral or intravenous systemic administration, and long-term use may increase the risk of gastrointestinal, kidney, and even cardiovascular diseases (Maniar et al., 2018; Mao et al., 2021). In contrast, intra-articular injection can effectively improve the concentration of drugs in the joint, but there is also a phenomenon of excessive local microenvironment metabolism, resulting in short drug retention time and it is thus difficult to achieve the expected therapeutic effect (Li G. et al., 2023; Kawaguchi, 2024). Surgical treatment, commonly used clinical surgical protocols include arthroscopic debridement, osteotomy and joint replacement, etc., is generally suitable for advanced bone and joint patients (Marx, 2008; Price et al., 2018; Pacheco-Brousseau et al., 2024). However, the surgical treatment is expensive and difficult to deal as a series of postoperative complications often happened (Canizares et al., 2021; Li H. et al., 2024).

In view of the challenges in the current clinical treatment of OA and the limitations of therapeutic means, many researchers have turned their attention to the development of biomedical remediation materials (Li et al., 2023b; Kumar et al., 2024). As a three-dimensional cross-linked polymer network biomaterial, hydrogels have been widely used in many fields such as tissue engineering (Lai et al., 2024), regenerative medicine, drug delivery and disease diagnosis due to their hydrophilic properties, controllable mechanical properties, excellent biodegradability and biocompatibility (Cao et al., 2021; Gutierrez et al., 2022; Zhao et al., 2023; Yuan et al., 2025). By using hydrogels as carriers of therapeutic drugs, researchers have achieved significant benefits in cartilage regeneration, inflammation alleviation and immune regulation of OA (Enayati et al., 2024; Ma et al., 2024; Zhang S. et al., 2024). Therefore, hydrogels are a potential biomedical material in the treatment of OA (Xue et al., 2022).

In the past, hydrogels were usually divided into natural hydrogels and synthetic hydrogels according to the different sources (Catoira et al., 2019). Natural sources of hydrogels include polysaccharide hydrogels (such as hyaluronic acid, chondroitin sulfate, chitosan, alginate, xanthan gum, heparin and fibroin, etc.) and polypeptide hydrogels (such as collagen, elastin, fibrin, gelatin and silk protein, etc. (Xu et al., 2024; Yadav et al., 2024). Synthetic hydrogels include poly (lactic-co-glycolic acid (PLGA), polylactic acid (PLA), polycaprolactone (PCL), polyethylene glycol (PEG), polyvinyl alcohol (PVA), etc., (Ahmed, 2015; Ho et al., 2022; Bramhe et al., 2024). At present, with the development and application of various new hydrogels, they have evolved from simple natural or synthetic hydrogels to hybrid intelligent responsive hydrogels with multiple characteristics to achieve accurate release (Tian and Liu, 2023; Liu L. et al., 2024). Smart responsive hydrogel is selected as a controlled drug release system for the treatment of osteoarthritis, which can respond to exogenous stimuli (light, ultrasound, pressure and magnetic field, etc.) or endogenous stimuli (temperature, enzyme, pH, ROS, etc.) to achieve controlled drug release, which has achieved remarkable results in the treatment of osteoarthritis (Jiang and Zhang, 2023).

**SCHEME 1:**
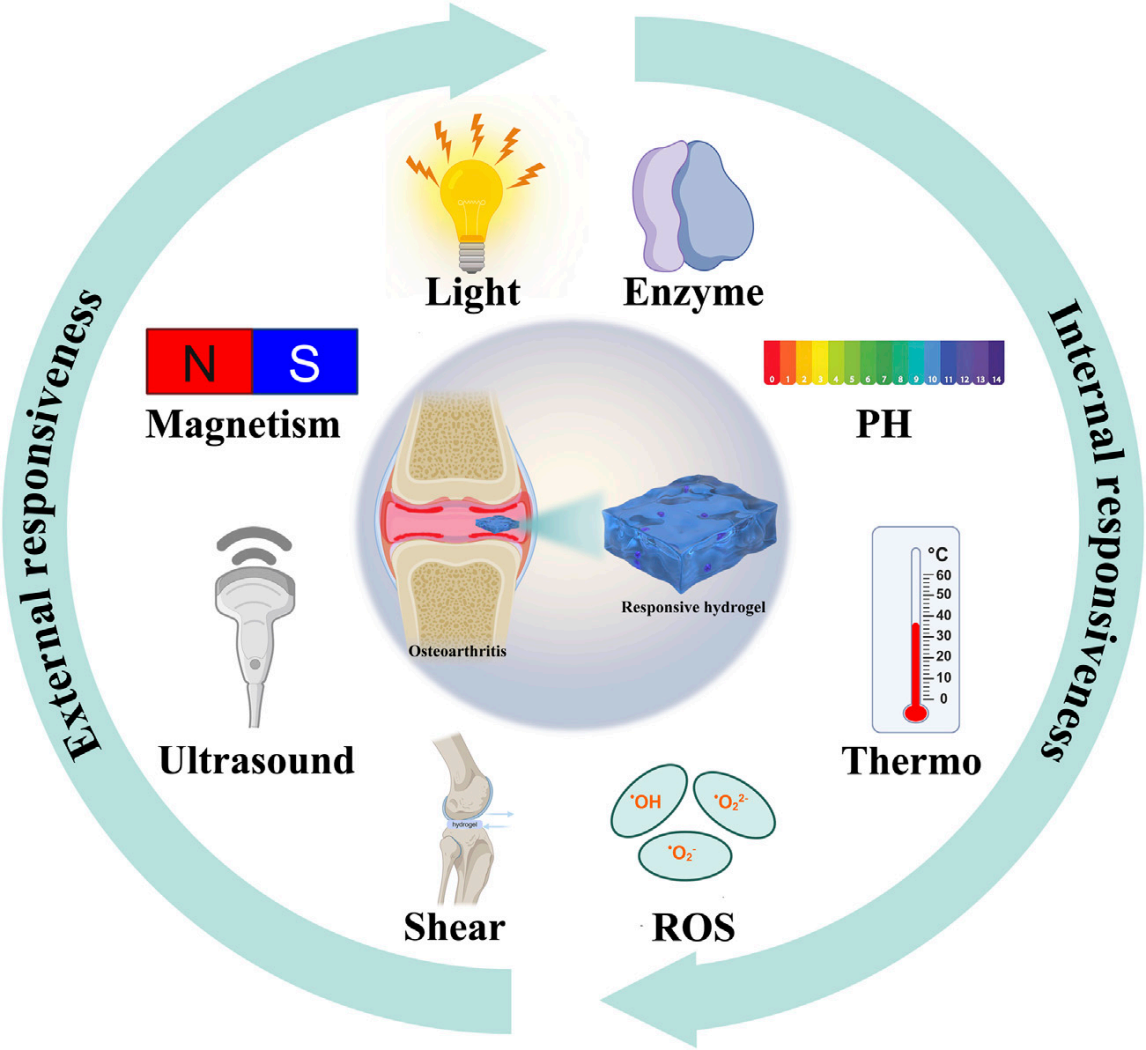
Multiple smart responsive hydrogels for the treatment of osteoarthritis.

**FIGURE 1:**
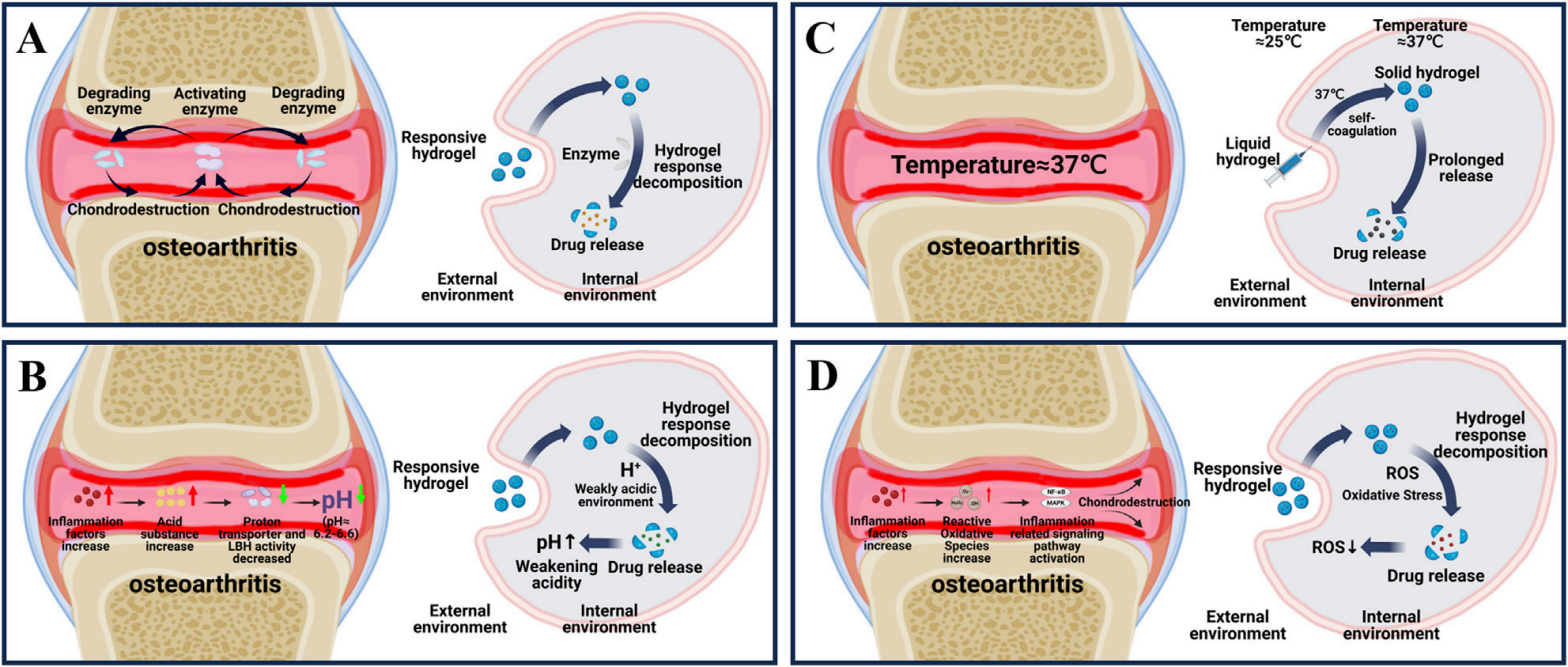
Principle of endogenous responsive hydrogels. **(A)** Changes in internal enzymes during osteoarthritis and the process by which enzyme responsive hydrogels release drugs in response to internal reactions. **(B)** Changes in internal PH during osteoarthritis and the internal response of PH-responsive hydrogels to release drugs. **(C)** Internal temperature during osteoarthritis and the process by which temperature-responsive hydrogels respond to self-coagulation and release drugs internally. **(D)** Changes in internal ROS during osteoarthritis and the process of drug release in response to ROS response hydrogels.

**FIGURE 2:**
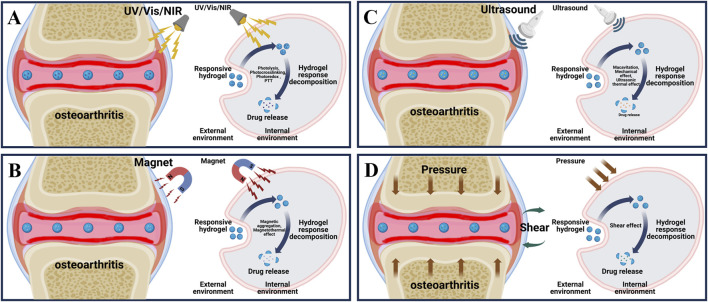
Principle of exogenous responsive hydrogels. **(A)** External light stimulation of the lesion area of osteoarthritis and the process of releasing drugs in response to external light stimulation of light responsive hydrogel. **(B)** External magnetic stimulation of the lesion area of osteoarthritis and the process of releasing drugs in response to external magnetic stimulation of magnetically responsive hydrogels. **(C)** External ultrasonic stimulation of the lesion area of osteoarthritis and the process of releasing drugs in response to external ultrasonic stimulation of the ultrasound-responsive hydrogel. **(D)** External shear stimulation in the lesion area of osteoarthritis and the process of drug release in response to external shear stimulation of shear-responsive hydrogel.

This review focuses on the latest applications of stimulus-responsive smart hydrogels in osteoarthritis (Scheme 1) and it will be introduced from the two aspects of endogenous (Figure 1) and exogenous (Figure 2) responsive hydrogels.

## 2 Internal responsive hydrogel

### 2.1 Enzyme responsive hydrogel

In recent years, the study of enzymes and their catalytic reactions has become a new hotspot (Buller et al., 2023). Due to the high selectivity, substrate specificity and good catalytic performance of enzyme-catalyzed reaction, enzyme-responsive hydrogels have more irreplaceable advantages compared with other biomaterials (Arcus and Mulholland, 2020; Sobczak, 2022; Reisenbauer et al., 2024). However, enzyme responsive hydrogels must also meet the following conditions: 1. The hydrogel network must be bound to a substrate with a high affinity to the enzyme, 2. The enzymatic reaction interacting with the enzyme must cause a significant change in the properties of the hydrogel, resulting in biodegradation or a change in structure that releases the loaded drug (Sobczak, 2022; Xiao and Huang, 2024).

As shown in Figure 1A, osteoarthritis is accompanied by an overproduction of a series of enzymes that cause material degradation as the disease progresses (Coryell et al., 2021). The key enzymes are protein-degrading enzyme and activator enzyme. Degrading enzymes break down the extracellular matrix (ECM), leading to a reduction in the hyaline cartilage matrix, which in turn roughens the articular cartilage and creates mechanical friction when it moves (Arra and Abu-Amer, 2023). The activation of enzyme can further enhance the activity of degrading enzyme in articular cartilage through positive feedback, and aggravate the destruction of cartilage (Arra and Abu-Amer, 2023). Therefore, targeting the overproduction of protein-degrading enzymes is the key to treating OA. Among various degrading enzymes, MMP and ADAMTS, as typical degrading enzymes in osteoarthritis, have been fully studied and targeted to develop enzyme-responsive hydrogels for the treatment of osteoarthritis (Table 1) (Cho et al., 2021; Mukherjee and Das, 2024; Wang et al., 2024a).

**TABLE 1:** Endogenous stimulus-responsive smart hydrogels for osteoarthritis therapy.

Internal stimulus	Material components	Responsive segment	Bioactive agent	Effects	References
Enzyme	Oxidized hyaluronic acid (OHA), dexamethasone sodium phosphate (DSP), Type I collagen (Col-I)	The Schiff’s base between OHA and Col-I	DSP	1.Reduces joint swelling 2.Downregulates IL-6 and TNF-α expression controls inflammation	Yi et al. (2022)
	Methacrylate-modified sulfonated azocalix arene (SAC4A MA), MMP-13 sensitive peptide, hydroxychloroquine (HCQ), methacrylated	MMP-13 sensitive peptide	HCQ	1.Removes excess ROS 2.Inhibition of MMP-13, IL-6 and TNF-α expression to control inflammation 3.Reduces cartilage destruction	Zhou et al. (2023)
	Hyaluronic acid (HA-MA), MMP-13 sensitive peptide,4-arm poly (ethylene glycol)-vinyl sulfone (PEG-VS),kartogenin (KGN)	MMP-13 sensitive peptide	KGN	1.Reduces the expression of MMP13, IL-6, TNF-α, IL-4 and IL-10 to achieve anti inflammatory 2.Inducing macrophages to differentiate into M2 to achieve immune regulation 3. Promotes cartilage repair	Tianyuan et al. (2023)
	Chondroitin sulfate methacryloyl (ChsMA), GelMA, celecoxib (CLX), Liposomes	GelMA	ChsMA, CLX	1.Inhibits the expression of MMP-13, TNF-α and IL-6 controls inflammation 2.Increases the expression of Agg and Col2a1 to promote cartilage regeneration 3.Controls joint space stenosis and osteophytes to delay OA progression	Miao et al. (2024)
pH	Phosphate-containing dendrimer (G1NC5.HCl), HIF-2 small interfering RNA (siRNA), HAMA	Self-polymerizing phosphate-containing dendrimer (G1-NC5.HCl)	HIF-2 siRNA	1.Anchoring cartilage 2.Downregulates HIF-2 expression and inhibits apoptosis 3.Promotes Col-II and inhibits MMP-13 expression to repair cartilage	Chen et al. (2023b)
	Neobavisolflavone (NBIF), zeolite-imidazole framework (ZIF-8 MOFs), Polydopamine (PDA), HA, GelMA	ZIF-8 MOFs	NBIF	1.Upregulated expression of Col-II and Sox-9 promoted cartilage differentiation 2.Inflammatory immunoregulation induced macrophage differentiation to M2	Jiang et al. (2023)
	OHA, adipic dihydrazide-grafted HA (HA-ADH), Selenium nanoparticles (SeNPs)	Schiff base bonds between OHA and HA-ADH	SeNPs	1.Upregulation of GPX1 inhibits oxidative stress 2.Upregulation of Col-II and downregulation of MMP13 decreased cartilage destruction	Hu et al. (2023)
	V_2_C MXenzyme NS, metformin, dextran methacryloyl (DexMA)	Amide bond between metformin and V_2_C MXenzyme	Metformin	1.V_2_C MXenzyme NS enhances the mechanics and lubrication of hydrogels 2.Clears excess ROS and activates the Nrf2-ARE pathway protects chondrocytes 3.Upregulated expressions of Col-II, Acan and Sox9 promote cartilage repair 4.Inhibits chondrocyte pyroptosis	Zhang et al. (2024b)
Thermo	Poly (lactic-co-glycolic acid)−poly (ethylene glycol)−poly (lactic-co-glycolic acid) (PLGA-PEG-PLGA),IL-36Ra	PLGA-PEG-PLGA	IL-36Ra	1.Reduces cartilage wear and tear slows OA progression	Yi et al. (2023)
	Pluronic F-127(F-127), HA, hydroxytyrosol (Hyt)	F-127	Hyt	1.Resists oxidative stress and clears excess ROS 2.The inflammatory cytokines IL-6, IL-8 and TNF-α are downregulated to inhibit inflammation 3.Delay chondrocyte senescence to reduce cartilage destruction	Valentino et al. (2022)
	O_3,_ D-mannose, hydroxypropyl chitin (HPCH), perfluorotributylamine, fluorinated HA	HPCH	O_3_ D-mannose	1.Immunity regulates and inhibits oxidative stress 2.Inhibits VEGF expression and controls inflammation 3.Reduces cartilage destruction and osteophyte formation while promoting cartilage regeneration	Wu et al. (2024a)
	Poloxamer-407 and 188,platelet-rich plasma-derived exosomes (PRP-Exo)	Poloxamer-407 and 188	PRP-Exo	1.Achieves long-term sustained drug release 2.Upregulates expression of TGF-β, Col-II, ACAN and SOX9 proteins to promote BMSCs migration and cartilage differentiation 3.Inhibits the expression of inflammatory factors slowed down cartilage destruction	Zhang et al. (2022b)
	Rapamycin (RAPA), poloxamer-407	Poloxamer-407	RAPA	1.Immune regulation that induces the transformation of macrophages from M1 to M2 2.Controls inflammation to slow cartilage destruction	Chen et al. (2023a)
ROS	Methyl methacrylate (MMA), polyvinyl acetate (PVA), GLX351322 (GLX), polyethylene glycol ketone mercaptan (mPEG-TK)	mPEG-TK	GLX	1.Inhibition of NOX4 expression to clear excess ROS and control inflammation 2.Reduces ferroptosis in macrophages	Zhen et al. (2024)
	Methacrylate-modified sulfonated azocalix arene (MACA),methylprednisolone (MP), 2-methacryloxyethyl phosphorylcholine (MPC), polyethylene glycol-modified poly thioketal (PEG-4PTK)	PEG-4PTK	MP	1.Responds quickly and clears excess ROS and downregulate HIF-1α levels 2.Controls inflammation progression 3.Lubricates joints to reduce cartilage wear	Wang et al. (2024b)
	3-Aminophenylbor-onic acid modified hyaluronic acid, hydroxyl-containing PVA	Phenylboronic ester bonds between the phenylboronic acids and the hydroxyl group	—	1.Clears excess ROS and down-regulating inflammatory factor IL-6, TNF-α achieves anti-inflammatory effect 2.Lubricates joints to reduce cartilage wear	Lei et al. (2024)
	3-Aminophenylb-oronic acid (APBA), silk fbroin (SF), alcohol, PVA, interferon-γ (IFN-γ)	The dynamic bonds between APBA and PVA	IFN-γ	1.Inhibites chondrocyte senescence 2.Promotes ATP production and increases mitochondrial membrane potential to improve energy metabolism 3.Removes excess ROS	Liu et al. (2023b)

**FIGURE 3:**
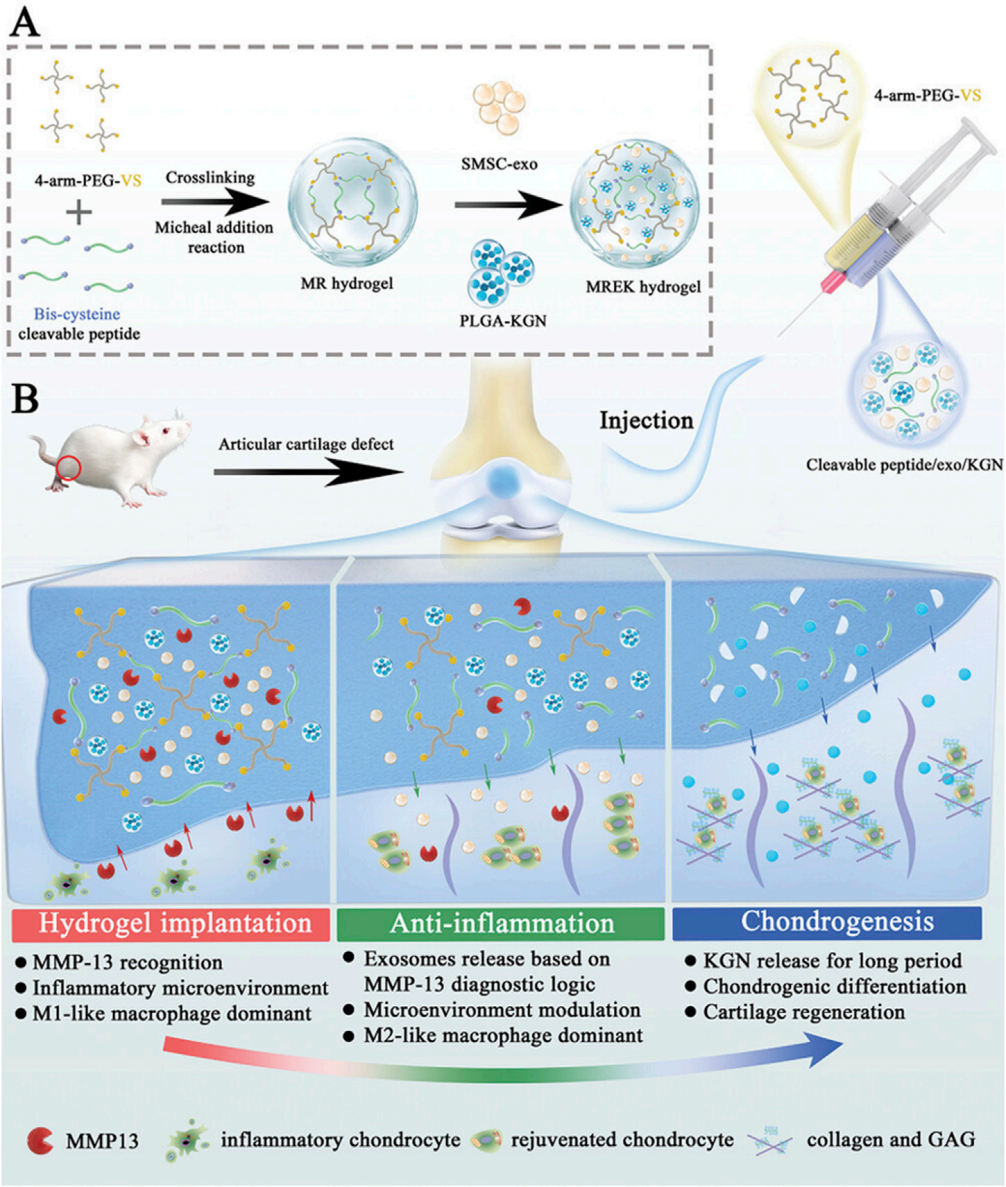
Preparation and working principle of MERK hydrogel. **(A)** Schematic diagram of the manufacture of MERK hydrogel. **(B)** Mechanism of action of MERK hydrogel after intraarticular injection. Reprinted with permission from (Tianyuan et al., 2023).

Yi et al. developed an MMP-responsive composite hyaluronic acid hydrogel (Col-OHA) for the treatment of osteoarthritis by cross-linking oxidized hyaluronic acid (OHA) with type I collagen and successfully loading the anti-inflammatory drug DSP based on the specific biodegradation of collagen by MMP (Yi et al., 2022). Col-OHA has good mechanical properties and injectable properties, and can self-decompose and slowly release DSP in response to MMP-1 *in vivo* and *in vitro* studies, downregulate the expression of inflammatory factors IL-6 and TNF-α, effectively control the progression of OA, and obtain good therapeutic effects. In contrast, Tong et al. prepared injectable MMP-13-responsive hyaluronic acid hydrogel microspheres by loading MMP-13 sensitive peptide and using microfluidic technology (HAM-SA@HCQ). In response to the microenvironment with high expression of MMP-13 in OA, the anti-inflammatory drugs HCQ and sulfonated azocaliarene (SACA) were specifically degraded and released. Excess ROS was removed from RAW264.7 macrophages stimulated by lipopolysaccharide (LPS) and IFN-γ, and the expression of HIF-1 was significantly reduced, and a good chondroprotective effect was achieved *in vivo* (Zhou et al., 2023). Similarly, Zhao et al., also based on MMP13 sensitive peptide, designed an MMP-13 responsive hydrogel (MREK) for early inflammation recognition and diagnosis of OA and coordination of cartilage repair (Figure 3A) (Tianyuan et al., 2023). MREK can respond to identify environments with high expression of MMP13, achieve early diagnosis and control of OA inflammation. As shown in (Figure 3B), MREK can effectively downregulate the expression of inflammatory factors (IL-6, TNF-α, IL-4 and IL-10) in the paracrine mediators of SMSCs, inhibit the inflammation of chondrocytes, and at the same time regulate the expression of genes (iNOS, CD86, Arg-1 and CD206) in macrophages, which plays a role in immune regulation. In addition, KGN, a supported chondro-inducing regenerative drug, is released through the response breakdown of MMP, achieving Col-II, ACAN and SOX9 were highly expressed.

**FIGURE 4:**
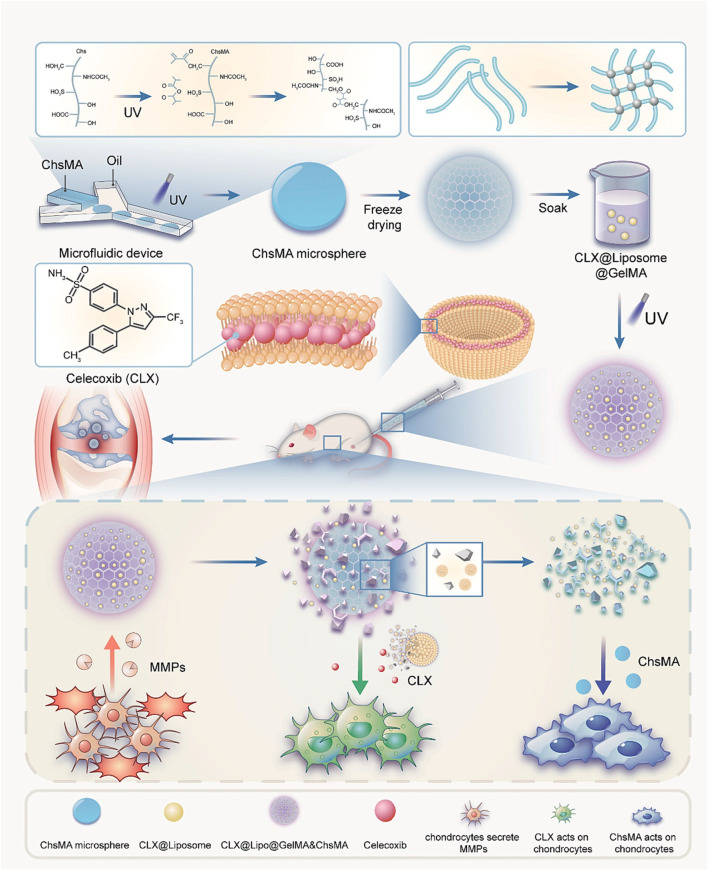
Preparation of ChsMA + CLX@Lipo@GelMA hydrogel and its working principle after injection into joint cavity. Reprinted with permission from (Miao et al., 2024).

Miao et al., prepared an injectable microenvironmentally responsive bilayer hydrogel microsphere (ChsMA + CLX@Lipo@GelMA) for OA joint lubrication, inflammation alleviation, and cartilage repair (Miao et al., 2024). As shown in (Figure 4), ChsMA + CLX@Lipo@GelMA is prepared from GelMA containing CLX liposomes as the shell and ChsMA microspheres as the core. The working principle is simply that GelMA, as a shell, can release CLX and liposomes in response to decomposition with MMP, which lubricate joints and remove excessive inflammatory factors such as TNF-α, IL-6 and MMP-13. The subsequent consumption of a large amount of CLX and liposomes exposed the ChsMA of the kernel, which was broken down into low-molecular weight chondroitin sulfate and chondroitin oligosaccharides, and increased the expression of chondrocyte protective factors Agg and Col2a1, thus achieving the effect of cartilage repair. *In vivo* and *in vitro* studies, ChsMA + CLX@Lipo@GelMA effectively controlled the progression of OA, achieving a combination of multi-layered response, precisely controlled drug release, multiple cartilage repair, inflammation clearance, and joint lubrication.

At present, enzymatic responsive hydrogels show good drug administration and therapeutic effect in the treatment of osteoarthritis, but there are still many limitations in their further application. At this stage, the changes in the types and quantities of pathogenic enzymes in the progression of osteoarthritis diseases are still in the preliminary research stage, and the changes in the activity of pathogenic enzymes after treatment have not been reported in detail. Therefore, the types of pathogenic enzymes that can be designed for enzyme-responsive hydrogels are relatively limited at present, and future research directions may focus on these aspects.

### 2.2 pH responsive hydrogel

Generally, in healthy joints, the pH of the microenvironment can be maintained in a stable state of 7.4–7.8 (Jiang and Zhang, 2023). As shown in Figure 1B, in the course of osteoarthritis, due to the increase of inflammatory factors, the increase of cell activity leads to the increase of cell oxygen consumption and the excessive production of acidic substances (McGarry et al., 2018). At the same time, the transport rate of proton transporters and lactate dehydrogenase a (LDHA), which maintain the pH of the microenvironment, is reduced, and excessive acidic substances cannot be excreted, resulting in a gradual transformation of the pH in the joint to a relatively weak acid (pH of about 6.2–6.6) (Zheng et al., 2021; Tan et al., 2022; Arra and Abu-Amer, 2023). In view of the pathological characteristics of osteoarthritis, researchers introduce acid reaction bonds (such as amino groups, carboxyl groups, etc.) or protonable chemical groups (such as o-esters, Schiff bases and vinyl ethers, etc.) into the pot-carrying gel to achieve the purpose of on-demand decomposition in the weak acid environment of osteoarthritis and precise release of drug therapy (Ding et al., 2022). At present, a variety of pH-responsive smart hydrogels have been used in the treatment of OA (Table 1).

**FIGURE 5:**
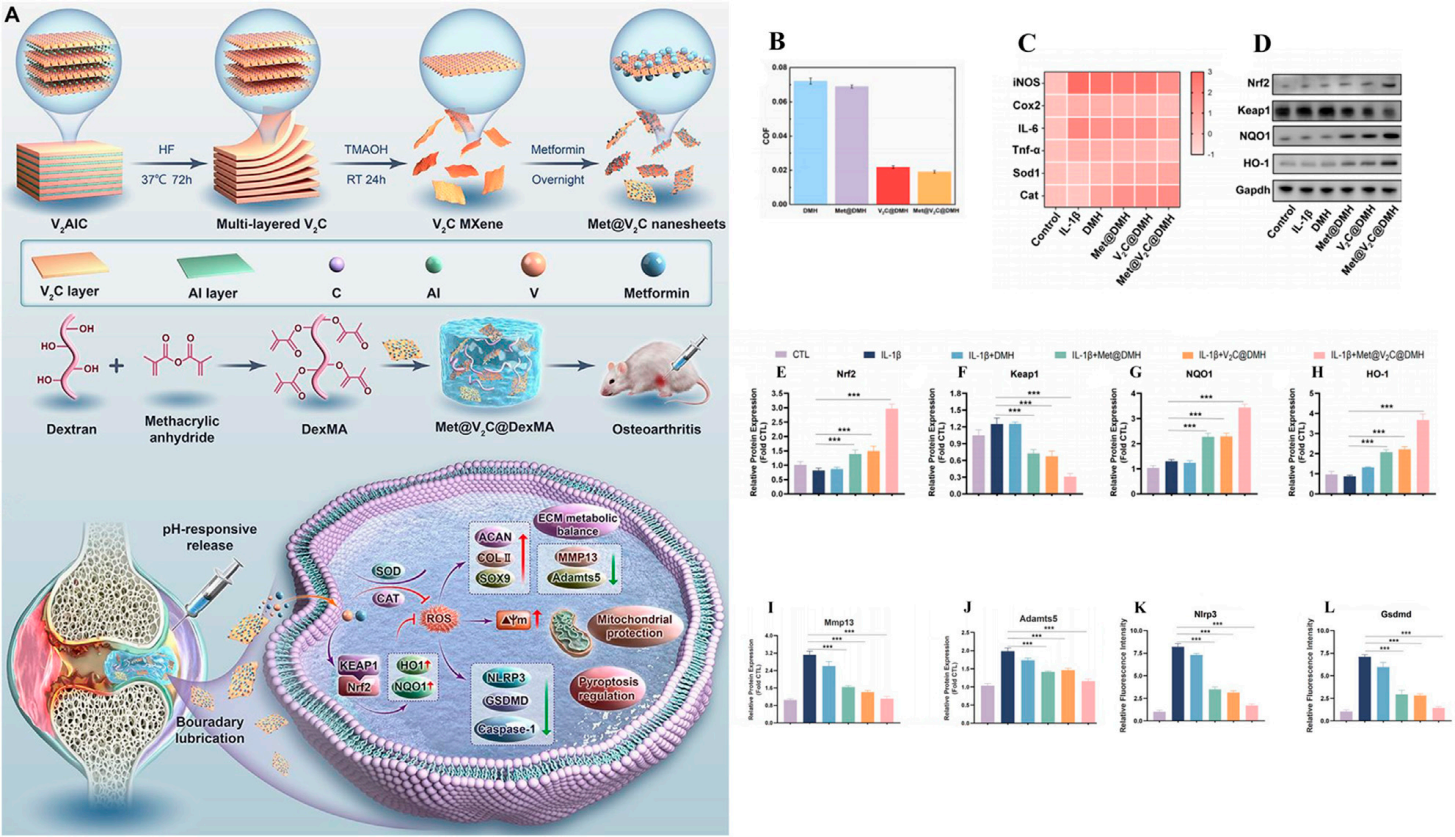
Preparation of hydrogel drug delivery system (Met@V_2_C@DMH) and *in vitro* study of therapeutic effect. **(A)** Preparation and working principle of Met@V_2_C@DMH. **(B)** Met@V_2_C@DMH lubrication effect. **(C)** Heat map of the relative mRNA expression levels of iNOS, Cox2, TNF-α, IL-6, Cat and Sod1 in chondrocytes of each group. **(D–H)** The expression of proteins in Nrf2-ARE antioxidant-related pathways in each group. **(I, J)** The protein expression of MMP13 and ADAMTS5 in chondrocytes of each group, **(K, L)** the expression of Nlrp3 and Gsdmd proteins associated with pyrodeath in chondrocytes in each group. (n = 3) (*p < 0.05, **p < 0.01, and ***p < 0.001). Reprinted with permission from (Zhang Z. et al., 2024).

As in Chen et al., inspired by the weakly acidic microenvironment of osteoarthritis, a pH-responsive injectable hydrogel microsphere for delivering siRNA of HIF-2 gene (associated with cartilage degradation) was prepared by protonation (HAMA/G1-NC5. HCl@siRNA) (Chen L. et al., 2023). When hydrogel microspheres were injected into the diseased joints, the protated phosphate-containing dendritic macromolecular derivatives would accurately anchor the injured cartilage in response to the weakly acidic microenvironment. It was found that Cy5.5 labeled siRNA could penetrate into the deep matrix of cartilage through fluorescence microscopy. In addition, radiological evaluation and histological analysis showed that siRNA delivery in diseased joints downregulated HIF-2 gene and inhibited MMP-13 protein expression, effectively slowed joint aging, promoted cartilage regeneration, reduced osteophytic formation, and improved OA joint stenosis. Based on ZIF-8 MOFs has a large specific surface area, high drug loading efficiency and pH sensitivity, Jiang et al., proposed a NBIF@ZIF-8/PHG hydrogel for the delivery of the anti-inflammatory drug NBIF for the treatment of OA (Jiang et al., 2023). NBIF@ZIF-8/PHG released a large amount of NBIF *in vitro* in a simulated OA microenvironment (pH 6.0), and co-incubated with bone marrow mesenchymal stem cells BMSCs and macrophages RAW264.7, respectively, to successfully test the role of NBIF in inducing the expression of Col-II and Sox-9 to promote cartilage regeneration and inflammatory immune regulation. *In vivo* studies, the high expression of Col-II and Agg in tissue staining analysis once again demonstrated its excellent cartilage repair effect. And the injectable hydrogel containing selenium nanoparticles proposed by Hu et al. (OHA/HA-ADH@SeNPs) achieves pH responsiveness of the joint environment through Schiff base bonds (Hu et al., 2023). SeNPs encapsulated in the hydrogel networks are released by Schiff base rupture in a weakly acidic inflammatory microenvironment to treat osteoarthritis by clearing excess ROS, inhibiting apoptosis and promoting cartilage repair. As shown in (Figure 5A), Zhao et al., developed an injectable hydrogel drug delivery system (Met@V_2_C@DMH) based on metformin and a novel nanosheet MXenzymes with multiple enzyme simulated activities such as oxide dismutase (SOD), catalase (CAT), and glutathione peroxidase (GPx) (Zhang Z. et al., 2024). The hydrogel utilizes the amide bond generated between metformin and MXenzyme to achieve pH response release. *In vivo* and *in vitro* studies have shown that Met@V_2_C@DMH can not only reduce the COF value of joint lubrication (Figure 5B), but also activate Nrf2-ARE antioxidation-related pathway (Figures 5D–H), downregulate the expression of inflammatory factors (TNF-α, COX2, iNOS and IL-6) (Figure 5C), MMP-13 and ADAMTS-5 proteins (Figures 5I, J). The chondrocyte damage was significantly reduced. In addition, Met@V_2_C@DMH downregulated the expression of Nlrp3 and Gsdmd proteins (Figures 5K, L), which proved that it had a certain anti-pyroptosis effect.

At present, pH-responsive hydrogels have achieved relatively considerable results in the treatment of osteoarthritis, but the changes of pH in the joint microenvironment after treatment lack long-term monitoring, and some chemical group changes in the joint have unknown effects. Therefore, the safety of future materials is also an aspect that cannot be ignored.

### 2.3 Thermo-responsive hydrogel

The temperature-responsive hydrogels have two aspects, which are different depending on the extra stimuli or not. In this section, the thermal-responsive hydrogels here are classified as endogenous reactions because the material itself is a fluid state at room temperature and can undergo morphological changes in response to internal temperature to self-aggregate into a gel state, where it is not affected by external stimuli. As for the other type of temperature-responsive hydrogel with external stimuli (such as magnetic force, ultrasound or light) mentioned below to influence temperature and cause conformation changes of hydrogels were classified as exogenous-responsive hydrogels. In this section, we pay attention to the endogenous responsive materials without extra stimulus.

The temperature of normal joints is usually slightly below 37°C (Xu et al., 2023). Based on the difference of internal and external environment of human joints, a thermo-responsive intelligent hydrogel for OA treatment was proposed (Li Y. et al., 2024). The core part is the successful loading of temperature-sensitive materials such as poly (n-isopropylacrylamide) (PNIPAM), poly (ninylisobutyramide) (PAMAM), CS, poloxam, pluronic F127, etc., into the hydrogel. As shown in Figure 1C, in the external room temperature environment (25°C), the thermosensitive smart hydrogel is a fluid state, and when injected into the internal environment of human joint tissue (37°C), the thermosensitive smart hydrogel can be sensitive to recognition, produce obvious physical and chemical property changes, and self-condense into a stable gel state (Singla et al., 2022; Rahmani et al., 2023). At the same time, the load drug is slowly released to achieve long-term OA treatment (Table 1) (Lv et al., 2023).

**FIGURE 6:**
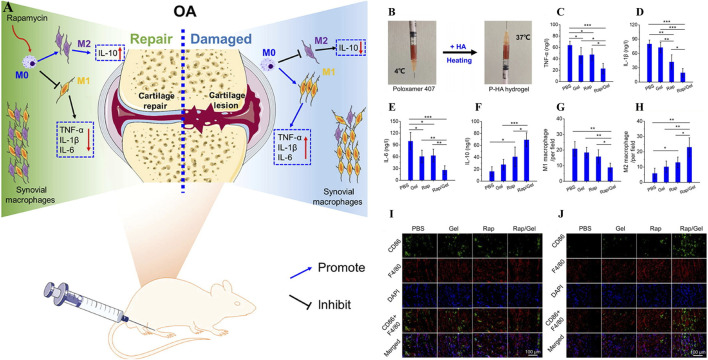
**(A)** Working principle of P-HA hydrogel. **(B)** Self-gelation of P-HA hydrogel. **(C–F)** Effects of P-HA hydrogel on synovial-related factors TNF-α, IL-1β, IL-6 and IL-10. **(G–H)** Quantitative analysis of M1 and M2 macrophages labeled by fluorescent staining in each group. **(I, J)** Immunofluorescence staining with CD86 or CD163 was added to F4/80 to label M1 or M2 macrophages in each group. (n = 3) (*p < 0.05, **p < 0.01, and ***p < 0.001). Reprinted with permission from (Chen B. et al., 2023).

As Yi et al., a temperature-sensitive poly PLGA-PEG-PLGA hydrogel (IL-36Ra@Gel) was successfully prepared (Yi et al., 2023). IL-36Ra, a receptor antagonist with IL-36, is used for OA control of inflammation and cartilage repair (Neurath, 2020). IL-36Ra@Gel is based on the fact that the temperature in the human joint is higher than room temperature. After injection into the joint, the hydrogen bond between PEG fragment and H_2_O molecule in the hydrogel in solution state becomes weak, and the hydrophobicity between PLGA fragment becomes stronger, resulting in the responsive cross-linking of the hydrogel from solution state to solid gel state, and the long-term release of IL-36Ra can be realized. By down-regulating MMP-13 and ADAMTS-5 and up-regulating the expression of Col-X and Agg, inflammation was inhibited and cartilage was protected in the treatment of OA. F-127 is a temperature-sensitive material based on hydrophobic polyethylene oxide (PEO) and hydrophobic polypropylene oxide (PPO) (PEO-PO-PEO) (Khaliq et al., 2023). Valentino et al. proposed a heat-response gel of chitosan nanoparticles based on F-127, HA and hydroxytyrosol Hyt (Hyt@tgel), which achieved intra-joint gelation through temperature changes after injection into joints, prolonged the release of polyphenol Hyt, and played a role in long-term inflammation alleviation (Valentino et al., 2022). Chen et al., prepared a heat-sensitive hydrogel (O_3_ NPs@MHPCH) using hydroxypropyl chitin (HPCH) for OA resistance to inflammation and cartilage protection (Wu H. et al., 2024). This composite hydrogel can self-gelatinize at 37°C, slowly release ozone (O_3_) and D-mannose, downregulate the expression of inflammatory factors IL-1, IL-6, TNF-α and iNOS, and promote the migration of chondrocytes, upregulate the expression of Col-II, and realize the self-repair of cartilage. Zhang et al., developed a temperature-sensitive hydrogel (Exo-Gel) that responds to the release of PRP-Exo for subtalar osteoarthritis (STOA) (Zhang Y. et al., 2022). Exo-Gel is temperature-controlled gelation via Pluronic-407 and 188, prolonging the release of Exo-Gel, which has anti-inflammatory effects and regulates cartilage regeneration. *In vitro* studies, Exo-Gel can maintain continuous drug release at 37°C for 1 month, and its promotion of proliferation and migration of mBMSCs was verified by CCK-8 and transwell scratch experiments. In addition, Exo-Gel can effectively inhibit the NF-κB inflammatory signaling pathway, decrease COLX and MMP13, and increase the expression of Col-II. *In vivo* studies, the animal model of STOA established by cutting ATFL/CFL was treated with Exo-Gel in the joint cavity for 8 weeks, and relatively complete and smooth cartilage was observed through histological staining analysis, which verified its excellent cartilage protection. Chen et al. also used the thermosensitive material Pluronic 407 and crosslinked with the HA and mTORC1 inhibitor rapamycin to prepare an injectable hydrogel (P-HA hydrogel) for the immune regulation of OA (Figure 6A) (Chen B. et al., 2023). In the incubation environment at 37°C, P-HA hydrogel can slowly release rapamycin in response to gelation (Figure 6B), downregulate the expression of pro-inflammatory factors TNF-α, IL-1β and IL-6, and upregulate the expression of anti-inflammatory factor IL-10 in macrophages induced by inflammation (Figures 6C–F). In addition, immunofluorescence staining suggested that P-HA hydrogel played a role in regulating the immune microenvironment by inducing macrophages to differentiate into M2 (Figures 6G–J). *In vivo* studies, P-HA hydrogel effectively inhibited synovial inflammation and mitigated cartilage destruction. It provides a new idea for the treatment of OA.

Thermo-responsive hydrogels greatly prolong the release of drugs through morphological changes, but inevitably affect the degradation rate of materials. In addition, the temperature of local joints often cannot maintain a constant physiological temperature for a long time due to the influence of external temperature, resulting in greatly reduced therapeutic effect. There is still a long way to go before thermo-responsive hydrogels can be widely used in the future.

### 2.4 ROS responsive hydrogel

In normal human joints, ROS, as a second messenger (Fang et al., 2024; Huang et al., 2024; Zhong et al., 2024), usually participates in the gene expression, signal transduction and cell cycle of chondrocytes, and plays an important role in maintaining the stability of the joint environment (Madreiter-Sokolowski et al., 2020; Hernansanz-Agustín and Enríquez, 2022). As shown in Figure 1D, in the pathogenesis of OA, high levels of ROS lead to the generation of intracellular oxidative stress, which then leads to the activation of signaling pathways related to inflammation and immune response (such as NF-κB and MAPK), damage the mitochondrial DNA and modified proteins of chondrocytes, and cause cell senescence and apoptosis (Bolduc et al., 2019; Ansari et al., 2020; Riegger et al., 2023). Therefore, the development of biological materials targeting oxidative stress is of great significance for OA treatment (Nakkala et al., 2021). At present, a variety of reactive oxygen species intelligent hydrogels designed in the study can deliver different drugs while removing ROS, and have achieved remarkable results in the treatment of OA (Table 1) (Zhang et al., 2023).

**FIGURE 7:**
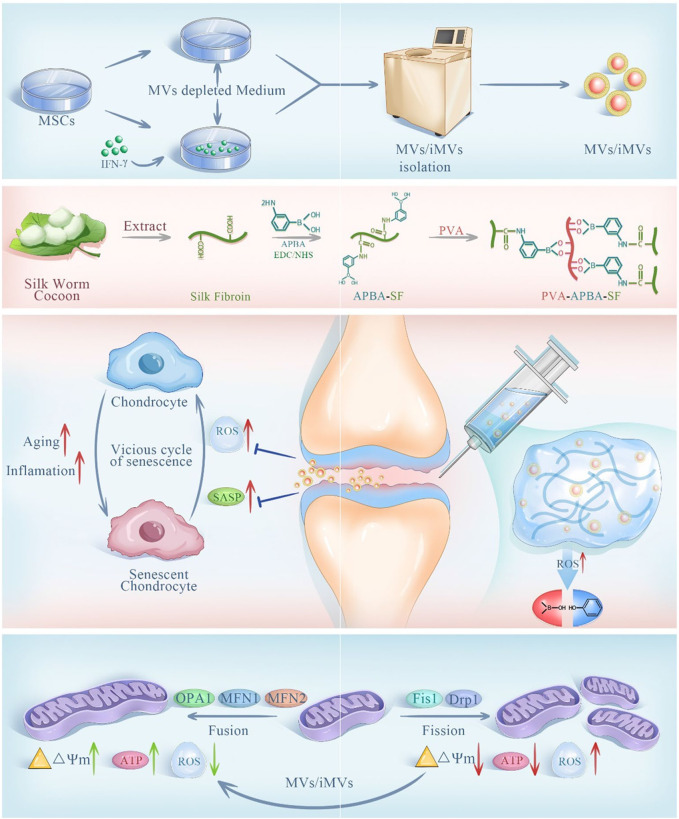
Preparation and principle of ROS responsive hydrogel @MVs/iMVs. Reprinted with permission from (Liu S. et al., 2023).

NADPH oxidase 4 (NOX4) is an enzyme protein used to produce ROS and is closely associated with iron death (Szanto, 2022). Based on the ROS responsiveness of polyethylene glycol ketone mercaptan (mPEG-TK), Jin et al., developed a ROS responsive hydrogel microsphere (mPEG-TK-GLX@PVA-MMA), which is composed of methyl methacrylate (MMA) modified PVA as the shell of the microsphere (Zhen et al., 2024). A novel selective NOX4 inhibitor, GLX, was prepared with mPEG-TK. mPEG-TK-GLX@PVA-MMA can release GLX in response to inflammation induced macrophages, and inhibit iron death by down-regulating the expression of Fe^2+^, C11, and JC-1 while clearing ROS. Zhao et al., developed an injectable microgel (MPTK-C@MP) for the delivery of MP for the treatment of OA in response to the properties of azo groups that can change reductase structure in anoxic environment (Wang et al., 2024b). MPTK-C@MP is prepared from the MACA, MPC and PEG-4PTK with MP supported base acrylate. *In vitro* studies, MPTK-C@MP successfully verified the ROS responsiveness by loading fluorescent chromogenic agent Rh123 in the incubation with chemical reducing agent SDT. In an environment of strong oxidative stress, it rapidly decomposing and releasing MP, regulating the immune microenvironment, down-regulating the expression of IL-6 and TNF-α in macrophages induced by inflammation, and eliminating excess ROS. At the same time, due to the presence of MPC, microgels can effectively reduce COF and provide joint lubrication. Finally, *in vivo* studies, MPTK-C@MP downregulates HIF-1α levels and enhances Col-II expression through potent anti-inflammatory and lubricating effects, demonstrating excellent cartilage repair effects. Lie et al. designed a bifocal injectable hydrogel (oHA-PBA-PVA), which was crosslinked by 3-aminophenylboric acid modified hyaluronic acid and hydroxy-containing polyvinyl alcohol (PVA) (Lei et al., 2024). It utilized the phenylborate ester bond formed by phenylboric acid and hydroxyl group to achieve ROS response while removing excess ROS. In addition, HPP gel has good rheological and mechanical properties, which can effectively downregulate COF to lubricate joints and protect cartilage. As shown in (Figure 7), Liu et al., aiming at excessive senescent cells in the pathogenesis of OA, prepared a ROS response hydrogel carrier through the dynamic bond formed between APBA modified SF and PVA, and intelligently released IFN-γ microbubbles (iMVs) (Liu S. et al., 2023). The expression of Ki67, P16, P21 and P53 in chondrocytes induced by inflammation was downregulated by stem cell therapy to delay cell senescence. At the same time, Hydrogel@MVs/iMVs also enhanced the expression of ATP and respiratory chain complex II, III and V, and provided a new treatment strategy for OA by improving mitochondrial function and enhancing cellular antioxidant capacity.

The balance of the redox system plays a crucial role in the stability of the biological environment. ROS responsive hydrogels will remove a large number of ROS during treatment, but it is doubtful whether the residual ROS can maintain the normal physiological activities of cells for a long time. In the future, how to balance the levels of physiological and pathological ROS may be the focus of optimization of such materials.

## 3 External responsive hydrogel

### 3.1 Light responsive hydrogel

Light is considered as a promising new energy source because of its good adjustability, high accuracy in space and time, and portability (Rapp and DeForest, 2020). Currently, researchers have developed a variety of light responsive hydrogels for OA treatment, among which common light sources include ultraviolet (UV) (200–400 nm), visible light (Vis) (400–700 nm), and near-infrared light (NIR) (700–1,300 nm) (Shen et al., 2021; Xing et al., 2022). As shown in (Figure 2A), by adjusting the direction, intensity, polarization, wavelength and frequency of light, the researchers accurately penetrate the tissue to produce different light stimuli, so that the photosensitive groups in the light responsive hydrogel are isomerized, resulting in a variety of effects such as photolysis, photocrosslinking, photoredox and photothermal triggering (photothermal therapy (PTT)), so as to achieve accurate and controlled release of loaded drugs (Table 2) (Li et al., 2019).

**TABLE 2:** Exogenous stimulus-responsive smart hydrogels for osteoarthritis therapy.

External stimulus	Material components	Responsive segment	Bioactive agent	Effects	References
Light	Anti-sense DNA sequence of the mRNA of IL-1β, gold nanorods (Au NRs),HA, NH_2_-PEG_4_-DBCO	NH_2_-PEG_4_-DBCO	Anti-sense DNA sequence of the mRNA of IL-1β	1.Photothermal responses and slow drug releases 2.Reduces oxidative stress and cartilage destruction	Chen et al. (2021)
	GelMA, Tyr, tris(2,2′-bipyridyl) dichlororuthenium (II), sodium persulfate (Ru/SPS)	Ru/SPS	GelMA-Tyr	1.Promotes cartilage adhesion and cartilage regeneration	Lim et al. (2020)
Magnetism	Lornoxicam (LOR), superparamagnetic iron oxide (SPION), HA, Synperonic™PE/F 127(PE/F127)	SPION	LOR	1.After intramuscular injection, the magnetic response gathered and gelated in the joint 2.Regulates motor activity 3.Inhibits the MAPK/ERK1 signaling pathway to control inflammation 4.Regulates the expression of RANKL/OPG to control bone destruction	Ibrahiem et al. (2023)
	GelMA, HAMA, Neodymium (NdFeB)	NdFeB	—	1. Mimics the diseased joint of osteoarthritis	Liu et al. (2024a)
	Gelatin, β-cyclodextrin (β-CD), magnetic, Fe_3_O_4_	Fe_3_O_4_	—	1.Induced BMSCs differentiation into cartilage	Liu et al. (2019)
	Acrylated β-cyclodextrins (Ac-β-CDs), gelatin, sodium alginate (Alg), dopamine hydrochloride (DA)	—	—	1.Promotes cartilage regeneration	Li et al. (2023d)
Ultrasound	Pluronic F-127, HA, Gelatin, hydrocortisone	Pluronic F-127	Hydrocortisone	1.Inhibits synovial inflammation and reduces cartilage destruction 2.Regulates motor behavior	Jahanbekam et al. (2023)
	Pentafluorophenol (PFP), dipalmitoyl phosphatidylcholine (DPPC), DSPE-PEG-2000-NH_2_, Stromal cell derived factor-1 α (SDF-1α)	—	SDF-1α	1.Activates the SDF-1-CXCR4 axis to enhance cell migration and cartilage regeneration	Liu et al. (2021)
	Carboxymethyl chitosan, Poly (vinyl alcohol) (PVA), Poly (lactide-*co*-glycolic acid) PLGA, chitosan (CS), KGN	—	KGN	1.Promotes cartilage regeneration	Yuan et al. (2021)
Shear	Aldehyde-modified HA (HA-CHO), adipic dihydrazide-modified HA (HA-ADH), celecoxib (CLX), phosphatidylcholine (HSPC) liposomes	Schiff base	CLX	1.Reduces coefficient of friction (COF) and lubricates joints 2.Downregulates MMP13 and ADAMTS5 and upregulates the expression of Col-II and AGG effectively reduces cartilage destruction	Lei et al. (2022a)
	Gelatin type A, HSPC liposomes, KGN, diclofenac sodiu (DS)	—	KGN, DS	1.Reduces COF and lubricates joints 2.Reduces joint inflammation and promotes cartilage regeneration 3.Reduces meniscus wea	Liu et al. (2023a)
	HAMA, HSPC liposomes, RAPA	—	RAPA	1.Reduces COF and lubricates joints 2.Scavengs excessive ROS and inhibits the expression of apoptosis-related genes LC3β and ATG5 provided chondrocyte homeostasis	Lei et al. (2022b)

**FIGURE 8:**
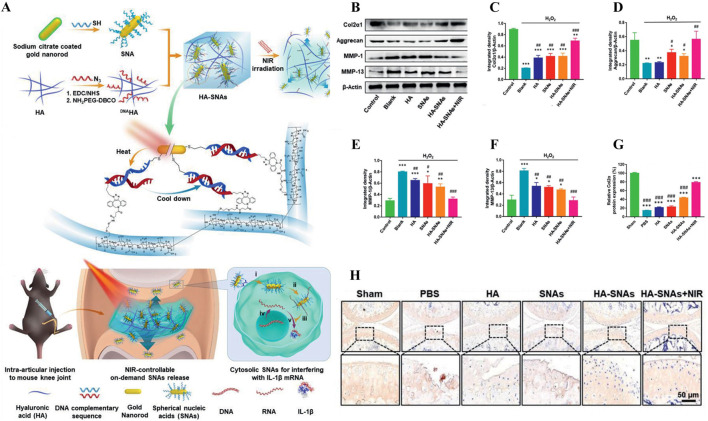
**(A)** Preparation and working principle of near-infrared photoresponsive hydrogel HA-SNAs, **(B–F)** Expression of Col2α1, Agg, MMP-1 and MMP-13 in cells treated with H_2_O_2_. **(G–H)** Immunohistochemical staining and quantification of joint Col2α1 in each group after treatment *in vivo*. n = 3, *p < 0.05, **p < 0.01, ***p < 0.001. Reprinted with permission from (Chen et al., 2021).

For example, the gene therapy of OA is to deliver the target gene to the specific binding site in the joint, and achieve controllable long-term treatment by regulating gene expression (Grol, 2024). IL-1 is a gene associated with the inflammatory progression of OA (Lee et al., 2021). As shown in Figure 8A, Chen et al. successfully prepared a NIR responsive hydrogel (HA-SNAs) for delivering IL-1 interfering oligonucleotides. It is prepared by hybridization of spherical nucleic acids (SNAs) made from Au NRs modified with antisense DNA sequence of IL-1 mRNA and Ha-grafted SNAs complementary DNA sequence (Chen et al., 2021). *In vitro* studies, Au NRs in HA-SNAs can respond to near-infrared light and convert it into heat energy, promote DNA unhelix decomposition and release interfering IL-1 mRNA molecules, downregulate the expression of MMP-1 and MMP-13 in H_2_O_2_-induced chondrocytes, and upregulate the expression of Col-II and Agg (Figures 8B–F). It has significant anti-inflammatory and cartilage protective effects. *In vivo* studies, HA-SNAs can effectively reduce cartilage surface erosion and deformation, and upregulate Col2*α* protein expression (Figures 8G, H), once again verify its good role in protecting cartilage and controlling the progression of OA. In addition, Lim et al. developed a bifocal tyramine methylacrylyl gelatin (GelMA-Tyr) based on the visible light response (Lim et al., 2020). It uses Ru/SPS as photoinitiators, which can respond to light crosslinking and adhere to cartilage with low biotoxicity, improve the expression of Col-II and effectively promote cartilage repair.

Although light has good controllability, it is inevitable that different types of light sources will cause damage to biological normal cells (such as thermal burn, carcinogenic transformation and apoptosis, etc.), in addition, different types of light sources have different penetrability to tissues and carry different energy (Jägerbrand and Spoelstra, 2023). For example, long-wavelength light or near-infrared light has strong tissue penetration, up to 10 cm deep in the tissue, but carries less energy. The tissue penetration of short-wavelength light or ultraviolet light is weak, sometimes only 10 mm below the tissue, but it carries more energy and the risk is relatively large. Visible light is somewhere in between. Therefore, screening suitable types of light sources and designing efficient photo-responsive hydrogels, balancing safety and therapeutic effect in deep tissue may be a greater challenge for future research.

### 3.2 Magnetism responsive hydrogel

Magnetic force is widely used in many medical fields such as magnetic resonance imaging (MRI), drug delivery and monitoring, enzyme quantitative measurement and magnetic hyperthermia therapy due to its strong tissue penetration ability and relatively free of harmful ionization effects (Li et al., 2020; Ramos-Sebastian et al., 2022). The magnetically responsive hydrogels are mostly composed of drug-carrying magnetic nanoparticle core (such as iron oxide, cobalt oxide and nickel oxide, etc.) and hydrogel shell, as shown in (Figure 2B), under the stimulation of the external constant magnetic field or alternating magnetic field (AMF), the magnetic nanoparticles will undergo structural changes, thereby controlling drug release and achieving precision treatment (Hao and Mao, 2023).

**FIGURE 9:**
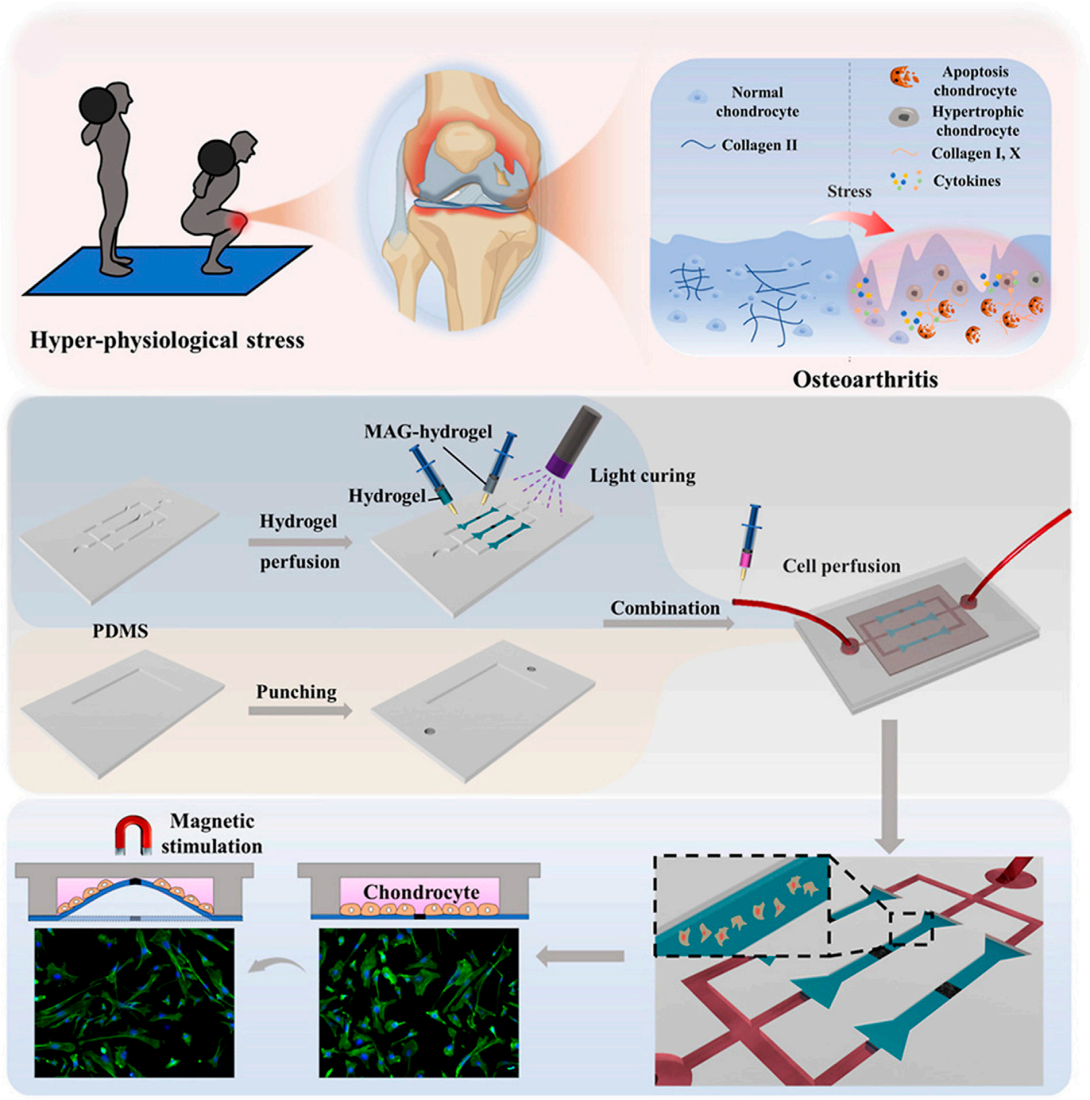
Preparation of magnetic hydrogels (NdFeB-GelMA-HAMA) and the working principle of simulating OA microenvironment change. Reprinted with permission from (Liu H. et al., 2024).

Based on the advantages of magnetic force, many magnetically responsive hydrogels for OA treatment have been developed for drug delivery (Table 2) (Li et al., 2021b). For example, Ibrahiem1 et al. envisioned a magnetic thermosensitive hydrogel (LSB) for intramuscular (IM) administration based on magnetic targeting and magnetothermal effects (Ibrahiem et al., 2023). In order to improve the bioavailability of the drug, they prepared LSB by loading the anti-inflammatory drug Lornoxicam (LOR) and superparamagnetic iron oxide nanoparticles (SPIONs) in a lipid bilayer gel and crosslinking it with a thermosensitive hydrogel containing Synperonic™PE/F127 (PE/F127). It was found that after LSB was injected into the thigh muscle, magnetic targeted aggregation of LSB was achieved by applying an external magnetic field to the knee joint. In addition, due to the thermal effect of magnetic aggregation, LSB is responsive to gelation, resulting in a slow and long-term release of LOR, successfully inhibiting the MAPK/ERK1 signaling pathway related to OA inflammation and regulating the expression of RANKL/OPG, effectively easing the progression of OA. To develop a new dynamic 3D model of OA *in vitro*. As show in Figure 9, Liu et al. designed a novel chip cartilage that uses a magnetic field to achieve dynamic cyclic stress regulation for simulating OA microenvironment changes (Liu H. et al., 2024). Researchers prepared a magnetic hydrogel (NdFeB-GelMA-HAMA) by combining the strong magnetic material NdFeB with GelMA-HAMA hydrogel, and cultured chondrocytes on this hydrogel. Through magnetic stimulation, the NdFeB-GelMA-HAMA responsive deformation is realized to simulate the change of stress during joint motion. Studies have found that chondrocytes will produce abnormalities under excessive mechanical stress, resulting in the reduction of type II collagen and the secretion of a large number of inflammatory factors such as MMP-13 and ADMTS-5 (Li et al., 2021b). Therefore, magnetic mechanical conversion chip cartilage prepared by magnetic effect has a good prospect as an ideal OA model for disease research and drug development. Liu et al. developed a pulsed electromagnetic field as a novel magnetically responsive smart hydrogel for controlling the specific chondrogenic differentiation of MSCs (Liu et al., 2019). Magnetic hydrogels were synthesized by chemical crosslinking of gelatin with β-cyclodextrin and magnetic embedding of Fe_3_O_4_. The adhesion, growth and proliferation of MSCs grown on magnetic hydrogels under pulsed electromagnetic field increased rapidly, and cartilage formation increased. *In vitro* experiments, it showed upregulation of Col-II, Agg and SOX9 genes. It provides a new direction for OA cartilage repair. Li et al. formed a novel magnetohydrogel by photocrosslinking Ac-β-CDs and gelatin and supplementing with dopamine-functionalized alginate (Alg-DA) (Li Y. et al., 2023). Similarly, Alg-DA/Ac-β-CD/gelatin hydrogel can promote cartilage repair and regeneration under pulsed electromagnetic field (PEMF).

Because it is still difficult to establish a specific magnetic field outside the organism at this stage, and the impact of magnetic field on biosecurity is still unknown, the application of magnetic response hydrogels in osteoarthritis is still in the preliminary exploration stage, but with the progress of science and technology, it is believed that more new magnetic response materials will be developed and applied in the future.

### 3.3 Ultrasound responsive hydrogel

Ultrasound has a promising future in drug delivery, disease diagnosis and treatment due to its relatively simple operation, effective focused energy and deep penetration (Chu et al., 2023; Liang et al., 2024). Ultrasound at 3–20 kHz frequencies has been widely used in clinical orthopedic (Song et al., 2023). As shown in (Figure 2C), since ultrasound can produce a variety of stimuli through the effects of cavitation, changing pressure, promoting acoustic fluid flow and ultrasonic local heat accumulation, various ultrasonic responsive hydrogels are designed to respond to various stimuli respectively, decompose and release loaded drugs for OA repair treatment (Table 2) (Zhou et al., 2022).

**FIGURE 10:**
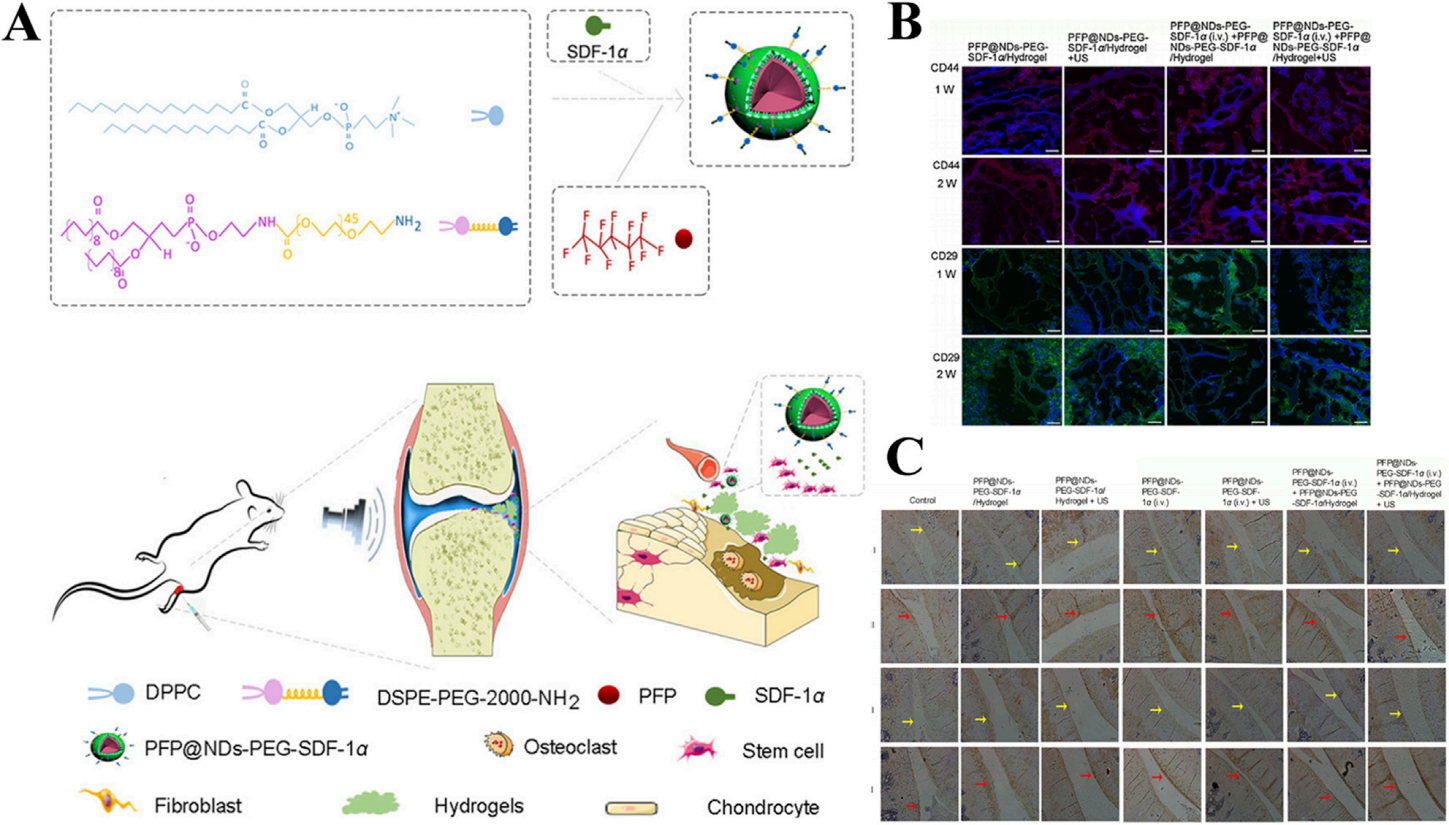
**(A)** Preparation and *in vivo* operation of ultrasonic responsive hydrogel (PFP@NDs-PEG-SDF-1α). **(B)** Expression of CD44 and CD26 in each group of rats after 1 and 2 weeks of treatment in PFP@NDs-PEG-SDF-1α. **(C)** type I and type II collagen expression after 6 and 12 weeks of treatment in PFP@NDs-PEG-SDF-1α in each group. Reprinted with permission from (Liu et al., 2021).

Jahanbekam et al. designed an ultrasonic thermal responsive hydrogel (MMH) made of Pluronic F-127, hyaluronic acid and gelatin based on the ultrasonic heat concentration effect, which successfully loaded hydrocortisone, a glucocorticoid drug, for the treatment of OA (Jahanbekam et al., 2023). *In vivo* and *in vitro* studies have found that liquid MMH can rapidly respond to temperature rise and gelate under ultrasonic waves at frequencies of 160 W and 35 kHz, achieving long-term controlled release of hydrocortisone, inhibiting OA synovial inflammation and easing cartilage degradation. SDF-1α is a factor closely related to the activation, mobilization, homing and migration of BMSCs (Li et al., 2023c). In order to achieve long-term effective release of SDF-1α, Liu et al. also prepared an ultrasound enhanced injectable chemotactic hydrogel (PFP@NDS-PEG-SDF-1α) based on the ultrasonic thermal effect for OA cartilage repair therapy (Figure 10A) (Liu et al., 2021). *In vitro* studies, PFP@NDS-PEG-SDF-1α hydrogel responded to ultrasonic gelation and promoted the migration of BMSCs based on the SDF-1-CXCR4 axis. In addition, *in vivo* studies, PFP@NDS-PEG-SDF-1α hydrogel can improve cell permeability and the expression of CD44, CD29 and Col-II under ultrasound (Figures 10B, C), promote the homing of BMSCs and repair cartilage defects at the same time, providing a new scheme for the treatment of OA. Yuan et al., combined KGN loaded PLGA hydrogel microspheres (MPs@KGN) with methyl chitosan oxidized chondroitin sulfate (CMC-OCS) hydrogel to prepare an ultrasonically responsive hydrogel for cartilage regeneration (CMC-OCS@MPs@KGN) (Yuan et al., 2021). The composite hydrogel can break under ultrasonic stimulation and quickly release high concentration of KGN, improve the expression of Col-II, and contribute to the repair of cartilage.

Although ultrasound can effectively penetrate many soft tissue structures in the body, in the joint, due to the obstruction of bone tissue, ultrasound is more or less weakened, affecting the therapeutic effect of ultrasonic responsive hydrogel. Therefore, improving the penetration of ultrasound in bone will be the next key breakthrough.

### 3.4 Shear responsive hydrogel

The bilateral cartilage of the normal joints of the human body has a smooth surface, and has very low shear friction under various physiological pressures, which can effectively maintain daily movement (Ristaniemi et al., 2024). Under the stimulation of the increase of external joint friction and shear, the articular cartilage wear will be caused, and the expression of cartilage degrading enzyme will increase the cartilage destruction, and then increase the joint friction again, and finally form a positive feedback leading to the generation of OA (Szabo et al., 2023). Hydrogels are widely used in bio-lubrication as three dimensional network structure hydrating materials, however, due to its relatively weak mechanical properties, it is easy to fail and can not achieve long-term lubrication effect (Baig et al., 2024). In response to this problem, as shown in (Figure 2D), researchers have developed a variety of shear-responsive hydrogels, which use the frictional shear of the joint to stimulate the hydrogels to deform and release drugs (Table 2). At the same time, the modification allows the hydrogels to be reassembled to form a long-term and effective lubricating hydration layer and effectively treat OA (Ma P. et al., 2022).

**FIGURE 11:**
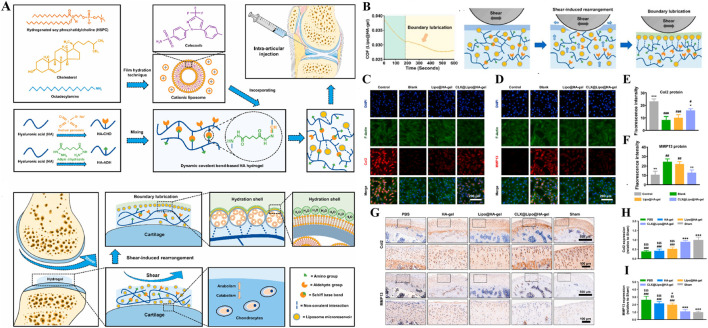
**(A)** Diagram of preparation and cartilage repair process of shear-responsive hydrogels (CLX@Lipo@HA-gels). **(B)** CLX@Lipo@HA-gels lubrication. **(C–F)** Immunofluorescence and quantitative characterization of Col2 and MMP13 after co-culture of chondrocytes *in vitro* with materials from each group. **(G–I)** Immunohistochemical staining and quantitative characterization of Col-II and MMP-13 in each group of tissue samples *in vivo*. n = 3, *p < 0.05, **p < 0.01, ***p < 0.001. Reprinted with permission from (Lei et al., 2022a).

As show in Figure 11A, Lei et al., constructed a shear-responsive drug-loaded hyaluronic acid hydrogel (CLX@Lipo@HA-gel) for OA boundary lubrication and inflammation alleviation based on the application of phospholipids in boundary lubrication (Lei et al., 2022a). CLX@Lipo@HA-gel uses dynamic Schiff base bonds between HA-CHO and HA-ADH to implement shear response. Under the action of shear force, CLX@Lipo@HA-gel dynamically decompositions and releases hydrogenated soybean phosphatidylcholine (HSPC) liposomes containing celercoxib (CLX). On the one hand, HSPC can rearrange with HA structure to form boundary layer to achieve stable and long-term lubrication. On the other hand, the release of CLX can effectively resist inflammatory factors and reduce the destruction of cartilage. *In vivo* and vitro studies, CLX@Lipo@HA-gel effectively decreased the expression of COF (Figure 11B) and regulated the expression of Col-II and MMP-13 (Figures 11C–I), which played a good role in cartilage protection. Liu et al. designed a self-lubricating and shear-response hydrogel (HKDG) for meniscus, considering that meniscus wear is closely related to cartilage destruction and OA formation (Liu L. et al., 2023). Under the stimulation of shear friction, the outer gel responds to rupture, releasing HSPC liposomes containing KGN and DS, providing a lubricated hydration layer while reducing inflammation in the joint microenvironment and promoting cartilage regeneration, effectively reducing the damage of meniscus and cartilage, and preventing the occurrence of OA. In addition, Lei et al. prepared an improved hydrogel microsphere (RAPA@Lipo@HMs) with a self-renewing boundary lubrication layer with better injectivity and lubricity (Lei et al., 2022b). The composite hydrogel microspheres encapsulated HAMA in the outer layer of cationic HSPC liposomes containing RAPA using microfluidic devices and photopolymerization techniques. Under shear friction, the hydrogel shell ruptures to release positively charged HSPC liposomes that target negatively charged cartilage and provide long-term boundary lubrication. In addition, during the formation of hydration layer, HSPC continuously released RAPA to achieve excessive ROS clearance and inhibit apoptosis of chondrocytes to maintain cell homeostasis. *In vivo* studies, the injection of RAPA@Lipo@HM into diseased joints effectively reduced cartilage wear and osteophytic formation, and played a good role in OA treatment. Overall, shear-responsive hydrogels provide good cartilage protection through long-term lubrication, providing a new way to treat osteoarthritis.

Due to the difficulty of controlling the shear force around the joint, the development of shear-responsive hydrogels is limited. At present, there are only a few research applications of shear-responsive hydrogels. Therefore, it is hoped that more scholars will pay attention to this aspect in the future and explore shear-related therapeutic discoveries.

## 4 Conclusion and prospect

Smart responsive hydrogels can deliver a variety of drugs to the specified space at the appropriate dose and within a specific time, reducing the adverse factors of drugs to normal tissues and greatly improving the bioavailability of drugs (El-Husseiny et al., 2022b). The intelligent responsive hydrogel is applied to the treatment of OA, which can adjust the release of drugs in response to various changes in the internal and external microenvironment of OA, realizing the purpose of precision treatment and achieving remarkable therapeutic effects (Zhang M. et al., 2022). In addition, the ingenious design of the researchers and the excellent natural chemical and physical properties of the hydrogels have led to the development of more hydrogels with multiple reaction effects and multiple drug loads, and intelligent responsive hydrogels are full of prospects on the road of future OA treatment (Gan et al., 2024).

There is no denying that smart reactive hydrogels have excellent therapeutic effects, but there is still a long way to go to expand their application for clinical use. First, although many intelligent-responsive hydrogels have been shown to have no significant biotoxic effects at both *in vivo* and *in vitro* study levels, most research methods are primarily based on lower-level animal models such as rats, mice, and rabbits (Kumi et al., 2024). Because there are still many differences between the structure of the human joint microenvironment with these animals (such as the thickness of the articular cartilage layer, the composition of the synovial fluid, etc.), it is doubtful whether the therapeutic effects and toxicity obtained in these animal models can be replicated in humans. Secondly, some external stimuli in response to exogenous hydrogels will inevitably cause damage to normal tissues (for example, ultrasound may damage DNA when it penetrates tissues, near-infrared light irradiation and magnetic concentration will cause thermal burns to surrounding local tissues, etc.) (El-Husseiny et al., 2022a). In addition, the human joint microenvironment is relatively complex, there are many uncertain interference factors, exogenous stimulation is difficult to achieve the ideal conditions for designing and preparing intelligent response hydrogels (Li M. et al., 2022). These results in misalignment or off-target control of drug release, would greatly reduce the effectiveness of treatment. Furthermore, many smart hydrogel drug delivery platforms are still in the initial stage of development. In order to achieve multiple therapeutic effects of osteoarthritis, it needs to spend a lot of energy and expense to build and decorate, and it is difficult to achieve the goal of commercialization in the short term (Rezakhani et al., 2024; Zöller et al., 2025). Moreover, different individual physiological states differ greatly, in order to achieve the desired therapeutic effect, individualized treatment is needed, which will undoubtedly increase the production cost again (Thang et al., 2023; Wu J. et al., 2024). Therefore, how to balance efficacy and cost may become a new problem affecting the development of intelligent response hydrogel research in the future. Finally, there is no perfect responsive hydrogel material. In the treatment of clinical diseases, it is necessary to comprehensively consider various factors such as disease characteristics, drug efficacy, physical and chemical properties, dosage and method of administration, and material cost, and keep pace with the times to continuously develop new responsive hydrogel systems, so that responsive hydrogel materials can truly move from the laboratory to clinical application.

At present, a variety of intelligent stimulus-responsive hydrogels have been successfully prepared and achieved excellent results in the treatment of OA. This review focuses on recent developments in stimulus-responsive hydrogels for the treatment of OA in the last 3 years since this article was written. The therapeutic effect of hydrogels with multiple responsive factors on drug delivery in the treatment of OA diseases and some existing problems were reviewed and discussed. Thus, this review contributes to an understanding of the most advanced research findings and corresponding challenges related to spirogenic stimulus-responsive hydrogels in OA therapy.
